# Methyl (2′-hydroxy­biphenyl-2-yl­oxy)acetate

**DOI:** 10.1107/S1600536809011362

**Published:** 2009-03-31

**Authors:** Muhammad Rabnawaz, Burhan Khan, Muhammad Raza Shah, Itrat Anis, Seik Weng Ng

**Affiliations:** a HEJ Research Institute of Chemistry, International Center for Chemical and Biological Sciences, University of Karachi, Karachi 75270, Pakistan; b Department of Chemistry, University of Malaya, 50603 Kuala Lumpur, Malaysia

## Abstract

The three independent mol­ecules of the title compound, C_15_H_14_O_4_, have similar orientations of their aromatic rings, the dihedral angles between the rings being 57.0 (1), 58.1 (1) and 58.2 (1)°. In each mol­ecule, the hydr­oxy group forms an intra­molecular hydrogen bond to the carbonyl O atom, forming an *S*(10) ring motif.

## Related literature

Only one of the two hydr­oxy groups of 2,2′-biphenyl-2,2′-diol underwent reaction to yield the mono-acetate title compound; a similar synthesis with *tert*-butyl bromo­acetate gave di-*tert*-butyl 2,2′-(biphenyl-2,2′-diyldi­oxy)diacetate; see: Ali *et al.* (2008 ▶).
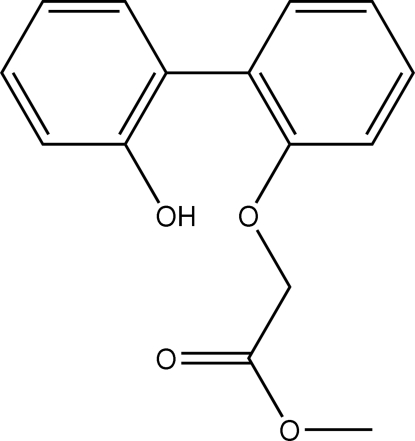


## Experimental

### 

#### Crystal data

C_15_H_14_O_4_
*M* *_r_* = 258.26
Monoclinic, 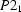

*a* = 10.9221 (2) Å
*b* = 7.5592 (1) Å
*c* = 23.3470 (4) Å
β = 98.465 (1)°
*V* = 1906.58 (5) Å^3^
*Z* = 6
Mo *K*α radiation
μ = 0.10 mm^−1^
*T* = 125 K
0.45 × 0.40 × 0.10 mm

#### Data collection

Bruker SMART APEX diffractometer
Absorption correction: none
13392 measured reflections
4639 independent reflections
4276 reflections with *I* > 2σ(*I*)
*R*_int_ = 0.025

#### Refinement

*R*[*F* ^2^ > 2σ(*F* ^2^)] = 0.031
*wR*(*F* ^2^) = 0.090
*S* = 1.05
4639 reflections
529 parameters
4 restraints
H atoms treated by a mixture of independent and constrained refinement
Δρ_max_ = 0.21 e Å^−3^
Δρ_min_ = −0.19 e Å^−3^

### 

Data collection: *APEX2* (Bruker, 2008 ▶); cell refinement: *SAINT* (Bruker, 2008 ▶); data reduction: *SAINT*; program(s) used to solve structure: *SHELXS97* (Sheldrick, 2008 ▶); program(s) used to refine structure: *SHELXL97* (Sheldrick, 2008 ▶); molecular graphics: *X-SEED* (Barbour, 2001 ▶); software used to prepare material for publication: *publCIF* (Westrip, 2009 ▶).

## Supplementary Material

Crystal structure: contains datablocks global, I. DOI: 10.1107/S1600536809011362/tk2406sup1.cif

Structure factors: contains datablocks I. DOI: 10.1107/S1600536809011362/tk2406Isup2.hkl

Additional supplementary materials:  crystallographic information; 3D view; checkCIF report

## Figures and Tables

**Table 1:** Hydrogen-bond geometry (Å, °)

*D*—H⋯*A*	*D*—H	H⋯*A*	*D*⋯*A*	*D*—H⋯*A*
O1—H1⋯O4	0.84 (1)	2.14 (2)	2.832 (2)	140 (3)
O5—H5⋯O8	0.84 (1)	2.12 (2)	2.856 (2)	145 (3)
O9—H9⋯O12	0.84 (1)	2.03 (2)	2.768 (2)	146 (3)

**Fig. 1.**
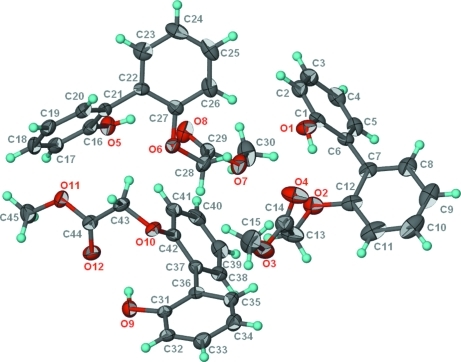
Thermal ellipsoid plot (Barbour, 2001) plot of the independent molecules of C14H14O4 at the 70% probability level; hydrogen atoms are drawn as spheres of arbitrary radius.

## supplementary crystallographic information

### Experimental

Potassium carbonate (0.69 g, 5 mmol) and 2,2'-biphenyl-2,2'-diol (0.93 mg, 5 mmol) in acetone (15 ml) were stirred for 20 minutes. Methyl 2-chloroacetate (0.22 g, 2 mmol) was added and the mixture was stirred at 323 K for 24 h. The solvent was removed and the residue was dissolved in a mixture of water (25 ml) and dichloromethane (25 ml). The two phases were separated and the aqueous layer was extracted with dichloromethane. The combined organic phases were dried and the solvent evaporated. The residue was dissolved recrystallized from dichloromethane (0.20 g, 30% yield).

### Refinement

Carbon-bound H-atoms were placed in calculated positions (C—H 0.93 to 0.99 Å) and were included in the refinement in the riding model approximation, with *U*(H) set to 1.2 to 1.5*U*_eq_(C). The hydroxy H-atoms were refined with a distance restraint of O–H 0.84±0.01 Å; their temperature factors were refined. In the absence of significant anomalous scattering effects, 3704 Friedel pairs were averaged in the final refinement.

### Crystal data

**Table:**

C_15_H_14_O_4_	*F*(000) = 816
*M**_r_* = 258.26	*D*_x_ = 1.350 Mg m^−^^3^
Monoclinic, *P*2_1_	Mo *K*α radiation, λ = 0.71073 Å
Hall symbol: P 2yb	Cell parameters from 6021 reflections
*a* = 10.9221 (2) Å	θ = 2.2–28.2°
*b* = 7.5592 (1) Å	µ = 0.10 mm^−^^1^
*c* = 23.3470 (4) Å	*T* = 125 K
β = 98.465 (1)°	Block, colorless
*V* = 1906.58 (5) Å^3^	0.45 × 0.40 × 0.10 mm
*Z* = 6	

### Data collection

**Table:**

Bruker SMART APEX diffractometer	4276 reflections with *I* > 2σ(*I*)
Radiation source: fine-focus sealed tube	*R*_int_ = 0.025
graphite	θ_max_ = 27.5°, θ_min_ = 0.9°
ω scans	*h* = −14→14
13392 measured reflections	*k* = −9→9
4639 independent reflections	*l* = −29→29

### Refinement

**Table:**

Refinement on *F*^2^	Primary atom site location: structure-invariant direct methods
Least-squares matrix: full	Secondary atom site location: difference Fourier map
*R*[*F*^2^ > 2σ(*F*^2^)] = 0.031	Hydrogen site location: inferred from neighbouring sites
*wR*(*F*^2^) = 0.090	H atoms treated by a mixture of independent and constrained refinement
*S* = 1.05	*w* = 1/[σ^2^(*F*_o_^2^) + (0.0585*P*)^2^ + 0.1469*P*] where *P* = (*F*_o_^2^ + 2*F*_c_^2^)/3
4639 reflections	(Δ/σ)_max_ = 0.001
529 parameters	Δρ_max_ = 0.21 e Å^−^^3^
4 restraints	Δρ_min_ = −0.19 e Å^−^^3^

### Fractional atomic coordinates and isotropic or equivalent isotropic displacement parameters (Å^2^)

**Table:**

	*x*	*y*	*z*	*U*_iso_*/*U*_eq_	
O1	0.71809 (15)	0.5002 (2)	0.08306 (7)	0.0281 (3)	
O2	0.91376 (15)	0.3602 (2)	0.17515 (7)	0.0308 (3)	
O3	0.96802 (18)	0.7736 (2)	0.24751 (8)	0.0403 (4)	
O4	0.89043 (18)	0.7116 (2)	0.15551 (8)	0.0415 (4)	
O5	0.29708 (14)	0.6042 (2)	0.28983 (7)	0.0257 (3)	
O6	0.50479 (13)	0.7375 (2)	0.23064 (6)	0.0246 (3)	
O7	0.64332 (15)	0.3191 (2)	0.21256 (7)	0.0325 (4)	
O8	0.45161 (15)	0.3890 (2)	0.22975 (7)	0.0326 (4)	
O9	0.78564 (13)	0.4140 (2)	0.49000 (6)	0.0245 (3)	
O10	0.62876 (12)	0.3744 (2)	0.37611 (6)	0.0221 (3)	
O11	0.35403 (13)	0.3858 (2)	0.44207 (6)	0.0255 (3)	
O12	0.54504 (14)	0.2888 (2)	0.47747 (7)	0.0296 (3)	
C1	0.69918 (19)	0.3221 (3)	0.07567 (8)	0.0224 (4)	
C2	0.5761 (2)	0.2690 (3)	0.06425 (9)	0.0252 (4)	
H2	0.5121	0.3547	0.0632	0.030*	
C3	0.5456 (2)	0.0929 (3)	0.05441 (9)	0.0284 (5)	
H3	0.4612	0.0583	0.0465	0.034*	
C4	0.6386 (2)	−0.0334 (3)	0.05609 (9)	0.0270 (5)	
H4	0.6182	−0.1548	0.0501	0.032*	
C5	0.7613 (2)	0.0199 (3)	0.06665 (9)	0.0246 (4)	
H5A	0.8245	−0.0666	0.0672	0.029*	
C6	0.79483 (19)	0.1973 (3)	0.07651 (8)	0.0215 (4)	
C7	0.92776 (19)	0.2461 (3)	0.08343 (9)	0.0244 (4)	
C8	0.9994 (2)	0.1989 (3)	0.04093 (10)	0.0322 (5)	
H8	0.9619	0.1358	0.0077	0.039*	
C9	1.1239 (2)	0.2421 (4)	0.04609 (13)	0.0405 (6)	
H9A	1.1714	0.2073	0.0170	0.049*	
C10	1.1781 (2)	0.3356 (4)	0.09370 (14)	0.0447 (7)	
H10	1.2628	0.3683	0.0966	0.054*	
C11	1.1116 (2)	0.3832 (3)	0.13741 (13)	0.0387 (6)	
H11	1.1500	0.4476	0.1701	0.046*	
C12	0.9875 (2)	0.3348 (3)	0.13249 (10)	0.0280 (4)	
C13	0.9554 (3)	0.4753 (3)	0.22167 (11)	0.0371 (6)	
H13A	1.0446	0.4547	0.2350	0.045*	
H13B	0.9099	0.4502	0.2544	0.045*	
C14	0.9352 (2)	0.6664 (3)	0.20314 (10)	0.0291 (5)	
C15	0.9453 (3)	0.9596 (4)	0.23748 (14)	0.0428 (6)	
H15A	1.0083	1.0285	0.2622	0.064*	
H15B	0.8631	0.9898	0.2467	0.064*	
H15C	0.9489	0.9870	0.1967	0.064*	
C16	0.28539 (18)	0.7837 (3)	0.29631 (9)	0.0220 (4)	
C17	0.26399 (19)	0.8417 (3)	0.35043 (9)	0.0263 (4)	
H17	0.2647	0.7595	0.3813	0.032*	
C18	0.24159 (19)	1.0195 (3)	0.35955 (10)	0.0289 (5)	
H18	0.2261	1.0585	0.3965	0.035*	
C19	0.24183 (19)	1.1403 (3)	0.31486 (10)	0.0268 (5)	
H19	0.2255	1.2618	0.3208	0.032*	
C20	0.26621 (18)	1.0815 (3)	0.26122 (9)	0.0231 (4)	
H20	0.2681	1.1648	0.2309	0.028*	
C21	0.28794 (17)	0.9034 (3)	0.25074 (8)	0.0200 (4)	
C22	0.30778 (18)	0.8449 (3)	0.19194 (8)	0.0206 (4)	
C23	0.2185 (2)	0.8801 (3)	0.14421 (9)	0.0266 (4)	
H23	0.1457	0.9427	0.1495	0.032*	
C24	0.2343 (2)	0.8250 (3)	0.08907 (9)	0.0325 (5)	
H24	0.1724	0.8496	0.0570	0.039*	
C25	0.3398 (2)	0.7348 (3)	0.08079 (9)	0.0305 (5)	
H25	0.3496	0.6953	0.0431	0.037*	
C26	0.4317 (2)	0.7011 (3)	0.12718 (9)	0.0264 (4)	
H26	0.5049	0.6398	0.1214	0.032*	
C27	0.41578 (19)	0.7577 (3)	0.18212 (9)	0.0217 (4)	
C28	0.6021 (2)	0.6175 (3)	0.22633 (10)	0.0278 (5)	
H28A	0.6669	0.6309	0.2604	0.033*	
H28B	0.6396	0.6445	0.1912	0.033*	
C29	0.5549 (2)	0.4293 (3)	0.22318 (9)	0.0250 (4)	
C30	0.6092 (3)	0.1327 (3)	0.20667 (12)	0.0405 (6)	
H30A	0.6684	0.0702	0.1863	0.061*	
H30B	0.5259	0.1221	0.1846	0.061*	
H30C	0.6102	0.0806	0.2452	0.061*	
C31	0.87045 (17)	0.4344 (3)	0.45300 (8)	0.0198 (4)	
C32	0.95512 (19)	0.5718 (3)	0.46605 (9)	0.0240 (4)	
H32	0.9501	0.6454	0.4986	0.029*	
C33	1.0468 (2)	0.6019 (3)	0.43176 (10)	0.0275 (5)	
H33	1.1029	0.6975	0.4404	0.033*	
C34	1.05647 (19)	0.4928 (3)	0.38491 (10)	0.0283 (5)	
H34	1.1191	0.5127	0.3614	0.034*	
C35	0.97378 (18)	0.3543 (3)	0.37285 (9)	0.0236 (4)	
H35	0.9818	0.2782	0.3412	0.028*	
C36	0.87894 (17)	0.3229 (3)	0.40565 (8)	0.0193 (4)	
C37	0.79318 (18)	0.1725 (3)	0.38900 (8)	0.0200 (4)	
C38	0.83849 (19)	0.0013 (3)	0.38485 (9)	0.0239 (4)	
H38	0.9249	−0.0195	0.3942	0.029*	
C39	0.7602 (2)	−0.1396 (3)	0.36738 (9)	0.0265 (4)	
H39	0.7925	−0.2554	0.3647	0.032*	
C40	0.6336 (2)	−0.1087 (3)	0.35387 (8)	0.0262 (4)	
H40	0.5794	−0.2045	0.3423	0.031*	
C41	0.5858 (2)	0.0602 (3)	0.35716 (9)	0.0236 (4)	
H41	0.4993	0.0801	0.3478	0.028*	
C42	0.66532 (18)	0.2002 (3)	0.37430 (8)	0.0201 (4)	
C43	0.50249 (18)	0.4079 (3)	0.38061 (8)	0.0236 (4)	
H43A	0.4491	0.3424	0.3498	0.028*	
H43B	0.4852	0.5357	0.3748	0.028*	
C44	0.47157 (18)	0.3521 (3)	0.43910 (9)	0.0224 (4)	
C45	0.3151 (2)	0.3513 (3)	0.49790 (9)	0.0308 (5)	
H45A	0.2274	0.3814	0.4961	0.046*	
H45B	0.3645	0.4234	0.5277	0.046*	
H45C	0.3273	0.2257	0.5076	0.046*	
H1	0.7905 (13)	0.520 (4)	0.0996 (12)	0.042 (8)*	
H5	0.335 (2)	0.578 (4)	0.2620 (9)	0.041 (8)*	
H9	0.726 (2)	0.350 (4)	0.4765 (14)	0.057 (10)*	

### Atomic displacement parameters (Å^2^)

**Table:**

	*U*^11^	*U*^22^	*U*^33^	*U*^12^	*U*^13^	*U*^23^
O1	0.0296 (8)	0.0196 (7)	0.0321 (8)	0.0012 (6)	−0.0059 (7)	−0.0010 (6)
O2	0.0364 (8)	0.0248 (8)	0.0291 (8)	−0.0027 (7)	−0.0023 (7)	−0.0023 (7)
O3	0.0474 (10)	0.0352 (10)	0.0352 (9)	−0.0064 (8)	−0.0041 (8)	−0.0110 (8)
O4	0.0560 (11)	0.0263 (9)	0.0356 (10)	−0.0014 (8)	−0.0153 (8)	0.0008 (7)
O5	0.0293 (8)	0.0234 (8)	0.0261 (8)	0.0014 (6)	0.0100 (6)	0.0045 (6)
O6	0.0228 (7)	0.0254 (8)	0.0252 (7)	0.0010 (6)	0.0024 (6)	−0.0064 (6)
O7	0.0353 (8)	0.0285 (8)	0.0353 (8)	0.0088 (7)	0.0108 (7)	−0.0002 (7)
O8	0.0338 (8)	0.0261 (8)	0.0405 (9)	−0.0011 (7)	0.0143 (7)	0.0006 (7)
O9	0.0245 (7)	0.0295 (8)	0.0198 (7)	−0.0006 (6)	0.0039 (6)	−0.0026 (6)
O10	0.0181 (6)	0.0238 (7)	0.0242 (7)	0.0004 (6)	0.0025 (5)	0.0043 (6)
O11	0.0221 (7)	0.0315 (8)	0.0238 (7)	0.0007 (6)	0.0068 (6)	−0.0009 (6)
O12	0.0261 (7)	0.0383 (9)	0.0240 (7)	0.0024 (7)	0.0020 (6)	0.0073 (7)
C1	0.0300 (10)	0.0207 (10)	0.0158 (9)	−0.0006 (8)	0.0009 (8)	0.0010 (7)
C2	0.0273 (10)	0.0279 (11)	0.0198 (10)	0.0021 (9)	0.0013 (8)	−0.0001 (8)
C3	0.0290 (11)	0.0322 (12)	0.0234 (10)	−0.0065 (9)	0.0023 (8)	0.0002 (9)
C4	0.0375 (12)	0.0209 (10)	0.0224 (10)	−0.0057 (9)	0.0033 (9)	−0.0012 (8)
C5	0.0332 (11)	0.0217 (10)	0.0188 (10)	0.0022 (9)	0.0036 (8)	0.0019 (8)
C6	0.0275 (10)	0.0208 (10)	0.0155 (9)	−0.0008 (8)	0.0002 (8)	0.0018 (8)
C7	0.0266 (10)	0.0192 (10)	0.0269 (10)	0.0027 (8)	0.0023 (8)	0.0080 (8)
C8	0.0363 (12)	0.0282 (11)	0.0330 (12)	0.0058 (10)	0.0083 (10)	0.0102 (10)
C9	0.0351 (13)	0.0334 (13)	0.0562 (16)	0.0079 (11)	0.0173 (12)	0.0196 (12)
C10	0.0292 (12)	0.0296 (13)	0.076 (2)	−0.0025 (10)	0.0090 (13)	0.0175 (13)
C11	0.0311 (11)	0.0216 (11)	0.0594 (16)	−0.0034 (10)	−0.0062 (11)	0.0084 (11)
C12	0.0300 (10)	0.0174 (9)	0.0348 (11)	−0.0003 (9)	−0.0015 (9)	0.0062 (9)
C13	0.0480 (14)	0.0317 (13)	0.0265 (11)	0.0004 (11)	−0.0118 (10)	−0.0005 (10)
C14	0.0269 (11)	0.0286 (12)	0.0300 (12)	−0.0030 (9)	−0.0016 (9)	−0.0043 (9)
C15	0.0384 (14)	0.0340 (14)	0.0566 (17)	−0.0058 (11)	0.0088 (12)	−0.0198 (12)
C16	0.0176 (9)	0.0238 (10)	0.0246 (10)	−0.0014 (8)	0.0028 (8)	−0.0001 (8)
C17	0.0228 (9)	0.0353 (12)	0.0216 (10)	−0.0054 (9)	0.0059 (8)	0.0011 (9)
C18	0.0224 (10)	0.0399 (12)	0.0258 (10)	−0.0074 (9)	0.0083 (8)	−0.0131 (9)
C19	0.0196 (9)	0.0272 (11)	0.0340 (12)	−0.0033 (8)	0.0050 (9)	−0.0108 (9)
C20	0.0189 (9)	0.0224 (10)	0.0269 (10)	−0.0008 (8)	−0.0001 (8)	−0.0013 (8)
C21	0.0160 (8)	0.0242 (10)	0.0196 (9)	−0.0006 (8)	0.0014 (7)	−0.0019 (8)
C22	0.0249 (9)	0.0170 (9)	0.0197 (9)	−0.0037 (8)	0.0023 (7)	−0.0002 (7)
C23	0.0323 (11)	0.0203 (10)	0.0251 (10)	−0.0007 (9)	−0.0025 (8)	0.0009 (8)
C24	0.0522 (14)	0.0225 (11)	0.0198 (10)	−0.0049 (10)	−0.0050 (9)	0.0022 (8)
C25	0.0533 (14)	0.0198 (10)	0.0192 (10)	−0.0063 (10)	0.0082 (9)	−0.0013 (8)
C26	0.0375 (12)	0.0195 (10)	0.0242 (10)	−0.0046 (9)	0.0108 (9)	−0.0030 (8)
C27	0.0273 (10)	0.0169 (9)	0.0209 (9)	−0.0049 (8)	0.0040 (8)	−0.0008 (7)
C28	0.0202 (10)	0.0314 (11)	0.0316 (11)	0.0023 (9)	0.0031 (9)	−0.0072 (9)
C29	0.0275 (10)	0.0301 (11)	0.0177 (9)	0.0020 (9)	0.0044 (8)	−0.0016 (8)
C30	0.0626 (17)	0.0237 (11)	0.0387 (13)	0.0098 (12)	0.0189 (12)	0.0034 (10)
C31	0.0180 (9)	0.0219 (9)	0.0182 (9)	0.0030 (8)	−0.0016 (7)	0.0005 (7)
C32	0.0228 (10)	0.0245 (10)	0.0232 (10)	0.0014 (8)	−0.0016 (8)	−0.0058 (8)
C33	0.0208 (10)	0.0276 (11)	0.0331 (11)	−0.0038 (9)	0.0007 (8)	−0.0063 (9)
C34	0.0227 (10)	0.0318 (12)	0.0316 (11)	−0.0034 (9)	0.0077 (9)	−0.0054 (9)
C35	0.0212 (9)	0.0260 (10)	0.0235 (10)	0.0014 (8)	0.0032 (8)	−0.0031 (8)
C36	0.0178 (9)	0.0192 (9)	0.0194 (9)	0.0017 (8)	−0.0021 (7)	0.0005 (7)
C37	0.0224 (9)	0.0222 (10)	0.0152 (9)	−0.0021 (8)	0.0027 (7)	0.0006 (7)
C38	0.0256 (10)	0.0251 (10)	0.0201 (9)	0.0017 (9)	0.0006 (8)	−0.0007 (8)
C39	0.0360 (11)	0.0219 (10)	0.0215 (10)	−0.0008 (9)	0.0035 (9)	−0.0003 (8)
C40	0.0358 (11)	0.0271 (11)	0.0150 (9)	−0.0108 (10)	0.0018 (8)	−0.0011 (8)
C41	0.0237 (10)	0.0284 (11)	0.0181 (9)	−0.0054 (8)	0.0013 (8)	0.0015 (8)
C42	0.0228 (9)	0.0237 (10)	0.0138 (9)	−0.0014 (8)	0.0028 (7)	0.0024 (8)
C43	0.0178 (9)	0.0290 (11)	0.0232 (9)	0.0023 (8)	0.0006 (7)	0.0060 (8)
C44	0.0220 (9)	0.0212 (10)	0.0239 (10)	−0.0022 (8)	0.0030 (8)	0.0004 (8)
C45	0.0336 (11)	0.0356 (12)	0.0258 (10)	−0.0028 (10)	0.0125 (9)	−0.0006 (9)

### Geometric parameters (Å, °)

**Table:**

O1—C1	1.369 (3)	C17—H17	0.9500
O1—H1	0.842 (10)	C18—C19	1.387 (3)
O2—C12	1.383 (3)	C18—H18	0.9500
O2—C13	1.414 (3)	C19—C20	1.391 (3)
O3—C14	1.322 (3)	C19—H19	0.9500
O3—C15	1.441 (3)	C20—C21	1.394 (3)
O4—C14	1.197 (3)	C20—H20	0.9500
O5—C16	1.373 (3)	C21—C22	1.488 (3)
O5—H5	0.843 (10)	C22—C23	1.394 (3)
O6—C27	1.388 (3)	C22—C27	1.400 (3)
O6—C28	1.412 (3)	C23—C24	1.388 (3)
O7—C29	1.326 (3)	C23—H23	0.9500
O7—C30	1.458 (3)	C24—C25	1.376 (4)
O8—C29	1.200 (3)	C24—H24	0.9500
O9—C31	1.365 (2)	C25—C26	1.388 (3)
O9—H9	0.839 (10)	C25—H25	0.9500
O10—C42	1.379 (3)	C26—C27	1.387 (3)
O10—C43	1.421 (2)	C26—H26	0.9500
O11—C44	1.321 (2)	C28—C29	1.511 (3)
O11—C45	1.453 (2)	C28—H28A	0.9900
O12—C44	1.210 (3)	C28—H28B	0.9900
C1—C2	1.391 (3)	C30—H30A	0.9800
C1—C6	1.406 (3)	C30—H30B	0.9800
C2—C3	1.383 (3)	C30—H30C	0.9800
C2—H2	0.9500	C31—C32	1.394 (3)
C3—C4	1.390 (3)	C31—C36	1.404 (3)
C3—H3	0.9500	C32—C33	1.389 (3)
C4—C5	1.387 (3)	C32—H32	0.9500
C4—H4	0.9500	C33—C34	1.387 (3)
C5—C6	1.400 (3)	C33—H33	0.9500
C5—H5A	0.9500	C34—C35	1.384 (3)
C6—C7	1.484 (3)	C34—H34	0.9500
C7—C8	1.397 (3)	C35—C36	1.396 (3)
C7—C12	1.402 (3)	C35—H35	0.9500
C8—C9	1.386 (3)	C36—C37	1.488 (3)
C8—H8	0.9500	C37—C38	1.394 (3)
C9—C10	1.376 (4)	C37—C42	1.404 (3)
C9—H9A	0.9500	C38—C39	1.389 (3)
C10—C11	1.384 (4)	C38—H38	0.9500
C10—H10	0.9500	C39—C40	1.391 (3)
C11—C12	1.393 (3)	C39—H39	0.9500
C11—H11	0.9500	C40—C41	1.386 (3)
C13—C14	1.515 (3)	C40—H40	0.9500
C13—H13A	0.9900	C41—C42	1.390 (3)
C13—H13B	0.9900	C41—H41	0.9500
C15—H15A	0.9800	C43—C44	1.514 (3)
C15—H15B	0.9800	C43—H43A	0.9900
C15—H15C	0.9800	C43—H43B	0.9900
C16—C17	1.389 (3)	C45—H45A	0.9800
C16—C21	1.400 (3)	C45—H45B	0.9800
C17—C18	1.388 (3)	C45—H45C	0.9800
			
C1—O1—H1	110 (2)	C24—C23—C22	121.1 (2)
C12—O2—C13	118.89 (19)	C24—C23—H23	119.4
C14—O3—C15	116.7 (2)	C22—C23—H23	119.4
C16—O5—H5	112 (2)	C25—C24—C23	120.0 (2)
C27—O6—C28	117.16 (16)	C25—C24—H24	120.0
C29—O7—C30	116.2 (2)	C23—C24—H24	120.0
C31—O9—H9	113 (2)	C24—C25—C26	120.4 (2)
C42—O10—C43	117.45 (16)	C24—C25—H25	119.8
C44—O11—C45	115.29 (17)	C26—C25—H25	119.8
O1—C1—C2	115.58 (19)	C27—C26—C25	119.3 (2)
O1—C1—C6	124.08 (19)	C27—C26—H26	120.3
C2—C1—C6	120.3 (2)	C25—C26—H26	120.3
C3—C2—C1	120.8 (2)	O6—C27—C26	123.53 (19)
C3—C2—H2	119.6	O6—C27—C22	115.08 (17)
C1—C2—H2	119.6	C26—C27—C22	121.37 (19)
C2—C3—C4	120.0 (2)	O6—C28—C29	110.71 (17)
C2—C3—H3	120.0	O6—C28—H28A	109.5
C4—C3—H3	120.0	C29—C28—H28A	109.5
C5—C4—C3	119.2 (2)	O6—C28—H28B	109.5
C5—C4—H4	120.4	C29—C28—H28B	109.5
C3—C4—H4	120.4	H28A—C28—H28B	108.1
C4—C5—C6	122.0 (2)	O8—C29—O7	126.0 (2)
C4—C5—H5A	119.0	O8—C29—C28	123.6 (2)
C6—C5—H5A	119.0	O7—C29—C28	110.38 (18)
C5—C6—C1	117.69 (19)	O7—C30—H30A	109.5
C5—C6—C7	118.99 (19)	O7—C30—H30B	109.5
C1—C6—C7	123.20 (19)	H30A—C30—H30B	109.5
C8—C7—C12	117.4 (2)	O7—C30—H30C	109.5
C8—C7—C6	120.0 (2)	H30A—C30—H30C	109.5
C12—C7—C6	122.56 (19)	H30B—C30—H30C	109.5
C9—C8—C7	121.5 (2)	O9—C31—C32	115.83 (18)
C9—C8—H8	119.2	O9—C31—C36	124.07 (18)
C7—C8—H8	119.2	C32—C31—C36	120.05 (18)
C10—C9—C8	119.4 (2)	C33—C32—C31	120.46 (19)
C10—C9—H9A	120.3	C33—C32—H32	119.8
C8—C9—H9A	120.3	C31—C32—H32	119.8
C9—C10—C11	121.3 (2)	C34—C33—C32	120.1 (2)
C9—C10—H10	119.3	C34—C33—H33	119.9
C11—C10—H10	119.3	C32—C33—H33	119.9
C10—C11—C12	118.7 (3)	C35—C34—C33	119.1 (2)
C10—C11—H11	120.6	C35—C34—H34	120.4
C12—C11—H11	120.6	C33—C34—H34	120.4
O2—C12—C11	124.5 (2)	C34—C35—C36	122.13 (19)
O2—C12—C7	113.84 (18)	C34—C35—H35	118.9
C11—C12—C7	121.5 (2)	C36—C35—H35	118.9
O2—C13—C14	110.53 (18)	C35—C36—C31	118.03 (18)
O2—C13—H13A	109.5	C35—C36—C37	118.44 (18)
C14—C13—H13A	109.5	C31—C36—C37	123.53 (17)
O2—C13—H13B	109.5	C38—C37—C42	118.14 (19)
C14—C13—H13B	109.5	C38—C37—C36	120.75 (18)
H13A—C13—H13B	108.1	C42—C37—C36	121.04 (18)
O4—C14—O3	125.4 (2)	C39—C38—C37	121.50 (19)
O4—C14—C13	124.1 (2)	C39—C38—H38	119.2
O3—C14—C13	110.4 (2)	C37—C38—H38	119.2
O3—C15—H15A	109.5	C38—C39—C40	119.1 (2)
O3—C15—H15B	109.5	C38—C39—H39	120.5
H15A—C15—H15B	109.5	C40—C39—H39	120.5
O3—C15—H15C	109.5	C41—C40—C39	120.8 (2)
H15A—C15—H15C	109.5	C41—C40—H40	119.6
H15B—C15—H15C	109.5	C39—C40—H40	119.6
O5—C16—C17	116.29 (19)	C40—C41—C42	119.49 (19)
O5—C16—C21	122.86 (19)	C40—C41—H41	120.3
C17—C16—C21	120.8 (2)	C42—C41—H41	120.3
C18—C17—C16	120.2 (2)	O10—C42—C41	124.37 (18)
C18—C17—H17	119.9	O10—C42—C37	114.63 (18)
C16—C17—H17	119.9	C41—C42—C37	120.9 (2)
C19—C18—C17	120.1 (2)	O10—C43—C44	111.40 (16)
C19—C18—H18	119.9	O10—C43—H43A	109.3
C17—C18—H18	119.9	C44—C43—H43A	109.3
C18—C19—C20	119.3 (2)	O10—C43—H43B	109.3
C18—C19—H19	120.4	C44—C43—H43B	109.3
C20—C19—H19	120.4	H43A—C43—H43B	108.0
C19—C20—C21	121.8 (2)	O12—C44—O11	125.55 (18)
C19—C20—H20	119.1	O12—C44—C43	124.49 (18)
C21—C20—H20	119.1	O11—C44—C43	109.95 (17)
C20—C21—C16	117.88 (18)	O11—C45—H45A	109.5
C20—C21—C22	120.02 (18)	O11—C45—H45B	109.5
C16—C21—C22	122.04 (18)	H45A—C45—H45B	109.5
C23—C22—C27	117.76 (18)	O11—C45—H45C	109.5
C23—C22—C21	120.09 (18)	H45A—C45—H45C	109.5
C27—C22—C21	122.14 (17)	H45B—C45—H45C	109.5
			
O1—C1—C2—C3	178.20 (19)	C22—C23—C24—C25	−0.3 (3)
C6—C1—C2—C3	1.0 (3)	C23—C24—C25—C26	−1.2 (3)
C1—C2—C3—C4	0.2 (3)	C24—C25—C26—C27	0.6 (3)
C2—C3—C4—C5	−1.2 (3)	C28—O6—C27—C26	−15.6 (3)
C3—C4—C5—C6	0.9 (3)	C28—O6—C27—C22	166.09 (18)
C4—C5—C6—C1	0.3 (3)	C25—C26—C27—O6	−176.90 (19)
C4—C5—C6—C7	−175.76 (18)	C25—C26—C27—C22	1.3 (3)
O1—C1—C6—C5	−178.19 (19)	C23—C22—C27—O6	175.67 (18)
C2—C1—C6—C5	−1.2 (3)	C21—C22—C27—O6	−2.9 (3)
O1—C1—C6—C7	−2.3 (3)	C23—C22—C27—C26	−2.7 (3)
C2—C1—C6—C7	174.66 (18)	C21—C22—C27—C26	178.75 (19)
C5—C6—C7—C8	54.1 (3)	C27—O6—C28—C29	−69.6 (2)
C1—C6—C7—C8	−121.7 (2)	C30—O7—C29—O8	2.0 (3)
C5—C6—C7—C12	−123.8 (2)	C30—O7—C29—C28	−178.2 (2)
C1—C6—C7—C12	60.4 (3)	O6—C28—C29—O8	−7.3 (3)
C12—C7—C8—C9	−1.8 (3)	O6—C28—C29—O7	172.94 (18)
C6—C7—C8—C9	−179.8 (2)	O9—C31—C32—C33	178.82 (19)
C7—C8—C9—C10	−0.9 (4)	C36—C31—C32—C33	1.3 (3)
C8—C9—C10—C11	1.9 (4)	C31—C32—C33—C34	−1.4 (3)
C9—C10—C11—C12	−0.1 (4)	C32—C33—C34—C35	0.1 (3)
C13—O2—C12—C11	14.5 (3)	C33—C34—C35—C36	1.4 (3)
C13—O2—C12—C7	−168.49 (19)	C34—C35—C36—C31	−1.4 (3)
C10—C11—C12—O2	174.1 (2)	C34—C35—C36—C37	179.1 (2)
C10—C11—C12—C7	−2.7 (3)	O9—C31—C36—C35	−177.18 (18)
C8—C7—C12—O2	−173.51 (19)	C32—C31—C36—C35	0.1 (3)
C6—C7—C12—O2	4.5 (3)	O9—C31—C36—C37	2.2 (3)
C8—C7—C12—C11	3.6 (3)	C32—C31—C36—C37	179.51 (19)
C6—C7—C12—C11	−178.4 (2)	C35—C36—C37—C38	56.0 (3)
C12—O2—C13—C14	78.3 (3)	C31—C36—C37—C38	−123.5 (2)
C15—O3—C14—O4	1.3 (4)	C35—C36—C37—C42	−120.9 (2)
C15—O3—C14—C13	−175.5 (2)	C31—C36—C37—C42	59.7 (3)
O2—C13—C14—O4	−0.2 (4)	C42—C37—C38—C39	−0.8 (3)
O2—C13—C14—O3	176.7 (2)	C36—C37—C38—C39	−177.68 (19)
O5—C16—C17—C18	−175.07 (19)	C37—C38—C39—C40	−0.1 (3)
C21—C16—C17—C18	1.7 (3)	C38—C39—C40—C41	0.6 (3)
C16—C17—C18—C19	−0.7 (3)	C39—C40—C41—C42	−0.2 (3)
C17—C18—C19—C20	−0.8 (3)	C43—O10—C42—C41	22.8 (3)
C18—C19—C20—C21	1.4 (3)	C43—O10—C42—C37	−159.91 (16)
C19—C20—C21—C16	−0.4 (3)	C40—C41—C42—O10	176.38 (18)
C19—C20—C21—C22	177.11 (18)	C40—C41—C42—C37	−0.8 (3)
O5—C16—C21—C20	175.43 (19)	C38—C37—C42—O10	−176.20 (16)
C17—C16—C21—C20	−1.1 (3)	C36—C37—C42—O10	0.7 (3)
O5—C16—C21—C22	−2.0 (3)	C38—C37—C42—C41	1.2 (3)
C17—C16—C21—C22	−178.58 (18)	C36—C37—C42—C41	178.14 (18)
C20—C21—C22—C23	−56.7 (3)	C42—O10—C43—C44	68.5 (2)
C16—C21—C22—C23	120.7 (2)	C45—O11—C44—O12	3.6 (3)
C20—C21—C22—C27	121.8 (2)	C45—O11—C44—C43	−175.28 (18)
C16—C21—C22—C27	−60.7 (3)	O10—C43—C44—O12	0.7 (3)
C27—C22—C23—C24	2.2 (3)	O10—C43—C44—O11	179.63 (17)
C21—C22—C23—C24	−179.24 (19)		

### Hydrogen-bond geometry (Å, °)

**Table:**

*D*—H···*A*	*D*—H	H···*A*	*D*···*A*	*D*—H···*A*
O1—H1···O4	0.84 (1)	2.14 (2)	2.832 (2)	140 (3)
O5—H5···O8	0.84 (1)	2.12 (2)	2.856 (2)	145 (3)
O9—H9···O12	0.84 (1)	2.03 (2)	2.768 (2)	146 (3)
